# Central obesity and blood pressure burden among Chinese children and adolescents with normal body mass index: a repeated-measures analysis of the China health and nutrition survey

**DOI:** 10.3389/fpubh.2026.1973651

**Published:** 2026-10-16

**Authors:** Xin Zhang, Lili Chen, Jiaqi Peng, Liuhui Su, Xiaoying Cao, Fa Chen

**Affiliations:** 1 Fujian Children’s Hospital (Fujian Branch of Shanghai Children’s Medical Center), College of Clinical Medicine for Obstetrics & Gynecology and Pediatrics, Fujian Medical University, Fuzhou, China; 2 Department of Epidemiology and Health Statistics, School of Public Health, Fujian Medical University, Fuzhou, China

**Keywords:** adolescents, blood pressure, central obesity, children, China health and nutrition survey, waist-to-height ratio

## Abstract

**Objective:**

Body mass index (BMI) is widely used to screen for pediatric adiposity, but it cannot differentiate fat distribution and fails to identify central fat accumulation among normal-weight youth. We aimed to evaluate whether central obesity was independently associated with high blood pressure among Chinese children and adolescents with normal BMI.

**Methods:**

We analyzed repeated examination data from 4,841 Chinese children and adolescents aged 7–17 years (7,192 complete-case observations) with normal BMI from the China Health and Nutrition Survey (1993–2011). Central obesity was defined as a waist-to-height ratio (WHtR) ≥ 0.50. The primary outcomes were above-normal blood pressure and high blood pressure at survey examination, classified using age-, sex-, and height-specific Chinese references. Population-average associations were estimated using generalized estimating equations (GEE) with robust standard errors. Standardized absolute risks, robustness checks, subgroup analyses, and exploratory longitudinal analyses were also performed.

**Results:**

Central obesity was present in 4.3% (323/7,469) of normal-BMI person-wave observations. In fully adjusted models, central obesity was associated with significantly higher odds of above-normal blood pressure (OR 1.43, 95% CI: 1.07–1.90; *p* = 0.016) and high blood pressure (OR 1.54, 95% CI: 1.07–2.21; *p* = 0.022). Standardized absolute risk differences were 5.1 and 3.5 percentage points, respectively. Sensitivity and subgroup associations were generally consistent across sex, urbanicity, and survey period. Exploratory longitudinal analyses (lagged, transition, and trajectory models) were directionally compatible with the primary findings but limited by sparse exposed observations.

**Conclusion:**

Among Chinese children and adolescents with normal BMI, central obesity was associated with a higher burden of blood pressure. Waist-based anthropometric assessment may complement BMI in identifying normal-weight youth with an unfavorable blood pressure profile, warranting confirmation in contemporary pediatric cohorts.

## 1 Introduction

Pediatric elevated blood pressure (BP) and hypertension have emerged as major global public health challenges, with current estimates indicating a worldwide pediatric hypertension prevalence of approximately 4 to 6%, and elevated BP affecting up to 10 to 15% of children and adolescents (1, 2). Elevated BP in early life is not a benign or transient phenomenon (3). Robust longitudinal evidence demonstrates that childhood blood pressure tracks strongly into adulthood (4), accelerating subclinical target-organ damage such as left ventricular hypertrophy, increased carotid intima-media thickness, and arterial stiffness (5, 6). In China, rapid epidemiological and nutritional transitions over recent decades have led to a marked increase in pediatric high blood pressure burden, making early identification and targeted screening a national public health priority (7, 8).

Body mass index (BMI) remains the universally recommended primary screening tool for pediatric overweight, obesity, and adiposity-related cardiometabolic risk in clinical guidelines (9). However, BMI is a measure of total body mass relative to height and cannot distinguish between fat mass and lean soft tissue, nor can it characterize body fat distribution (10, 11). Consequently, reliance on BMI alone creates a critical blind spot: children and adolescents with a normal BMI may still accumulate excess visceral and subcutaneous abdominal fat, a phenotype recognized as normal-weight central obesity or hidden adiposity (12, 13).

Abdominal fat accumulation is biologically more active than subcutaneous peripheral fat, releasing pro-inflammatory cytokines, causing low-grade systemic inflammation, and stimulating the renin-angiotensin-aldosterone system (RAAS) and sympathetic nervous system (SNS), all of which directly contribute to vascular dysfunction and blood pressure elevation (14–16). Waist-to-height ratio (WHtR) has been established as a simple, highly effective anthropometric indicator of central adiposity in youth (17, 18). A WHtR threshold of ≥0.50 (keep your waist circumference to less than half your height) provides a boundary that is largely independent of age, sex, and ethnicity, making it particularly advantageous for routine clinical and public health screening (10, 19).

Although cross-sectional studies have indicated links between central obesity and elevated BP across the general pediatric population (20, 21), evidence specifically focused on children and adolescents with normal BMI remains limited, geographically restricted, and largely based on single cross-sectional surveys. Cross-sectional evaluations cannot account for longitudinal intra-individual BP fluctuations or repeated observations over time. Furthermore, whether normal-weight central obesity independently confers elevated blood pressure burden in Chinese youth after comprehensive adjustment for pediatric growth trajectory, dietary intake, socioeconomic status, and urbanicity remains unclarified.

To address these knowledge gaps, we analyzed repeated-examination panel data from the China Health and Nutrition Survey (CHNS). Our primary objective was to investigate whether central obesity (WHtR ≥0.50) is independently associated with screening-level above-normal BP and high BP among Chinese children and adolescents aged 7–17 years who fall strictly within the normal-BMI range. We hypothesized that central obesity confers significant population-average odds of elevated blood pressure even among apparently normal-weight youth, highlighting the incremental utility of waist-based anthropometry in pediatric cardiovascular risk screening.

## 2 Materials and methods

### 2.1 Study design and data source

We conducted a secondary analysis of repeated examination data from the CHNS, an ongoing multistage, cluster-randomized household survey conducted in multiple Chinese provinces and megacities. Households and individuals were sampled using a multistage random-cluster design stratified by geographic region and urban–rural residence, with standardized anthropometric and blood-pressure measurements obtained at each examination visit by trained fieldworkers using uniform protocols (22, 23).

The present analysis used publicly available CHNS waves from 1993 through 2011. Earlier waves were excluded because pediatric waist-circumference measurement was not consistently available, precluding calculation of the primary exposure. Because participants could be examined at more than one wave, the data structure was that of repeated measurements clustered within individuals with unequal numbers of observations per participant and irregular intervals between waves.

The CHNS protocol was approved by the institutional review boards of the University of North Carolina at Chapel Hill and the Chinese Center for Disease Control and Prevention, and written informed consent was obtained from participants or their guardians. The present analysis used de-identified public-use data and required no additional ethical approval.

### 2.2 Eligibility criteria and analytic samples

Person-wave observations were eligible if the participant was aged 7–17 years at examination, had valid measurements of height, weight, and waist circumference, had at least two valid systolic and diastolic blood-pressure readings, and was classified as having normal BMI. Observations with implausible or missing anthropometric values, insufficient blood-pressure readings, or BMI outside the normal range were excluded. Restriction to the normal-BMI range was applied at the observation level, so a participant could contribute observations at some waves and not others.

Three nested analytic samples were defined *a priori* and are enumerated in Supplementary Table S1. The sample flow describes inclusion of available person-wave examination records rather than recruitment participation. The first-eligible-observation cohort (4,964 participants, of whom 222 had central obesity), used to describe participant characteristics. The repeated-observation descriptive sample (7,469 person-wave observations from 4,964 participants, including 323 central-obesity observations), used for descriptive and figure-based summaries; and the complete-case sample for the primary regression analysis (7,192 observations from 4,841 participants, including 317 central-obesity observations), restricted to observations with complete covariate information. The reduction from the descriptive sample to the complete-case sample was attributable to 198 observations missing dietary variables and 86 observations missing household income or household-size information, with partial overlap between these groups. Multiple imputation was not performed; instead, the potential influence of incomplete data was examined through the stepwise model progression and sensitivity analyses described below.

### 2.3 Anthropometric measurements and central obesity

Height was measured to the nearest 0.1 cm without shoes, weight to the nearest 0.1 kg in light clothing, and waist circumference to the nearest 0.1 cm at the midpoint between the lowest rib and the iliac crest at the end of normal expiration. BMI was calculated as weight (kg) divided by height (m) squared, and WHtR as waist circumference divided by height, both in the same units.

Normal BMI status was assigned using age- and sex-specific Chinese reference standards for school-aged children and adolescents; observations classified as wasting, overweight, or obesity were excluded from the risk set. The primary exposure, central obesity, was prespecified as WHtR ≥0.50, a threshold that is age- and sex-independent and widely applied in pediatric populations. Because BMI varies within the normal range and was modestly higher among participants with central obesity, BMI was retained as a continuous covariate in adjusted models so that the exposure contrast reflected differences in relative waist size rather than residual differences in overall body size. In a prespecified secondary specification, WHtR was modeled as a continuous variable scaled per 0.05-unit increment. For descriptive purposes, WHtR was additionally categorized as <0.40, 0.40-, 0.45-, and ≥0.50.

### 2.4 Blood-pressure measurement and outcome definitions

At each examination, up to three systolic and diastolic blood-pressure readings were obtained by trained observers using standardized mercury sphygmomanometers with age-appropriate cuff sizes, after the participant had rested in a seated position. Systolic and diastolic values were averaged across all valid readings at that visit, requiring a minimum of two readings for the observation to be eligible.

Blood-pressure status was classified using age-, sex-, and height-specific Chinese pediatric reference percentiles. Two co-primary binary outcomes were defined: (1) above-normal blood pressure, defined as mean systolic or diastolic blood pressure at or above the 90th percentile (i.e., high-normal blood pressure or high blood pressure combined); and (2) high blood pressure at survey examination, defined as mean systolic or diastolic blood pressure at or above the 95th percentile. Both outcomes represent examination-based screening phenotypes measured at a single visit and do not constitute a clinical diagnosis of hypertension, which requires elevated readings on separate occasions.

Prespecified secondary outcomes comprised mutually informative component phenotypes, high-normal blood pressure, isolated systolic high blood pressure, isolated diastolic high blood pressure, and combined systolic and diastolic high blood pressure, and continuous outcomes, namely mean systolic and diastolic blood pressure, the margin (in mm Hg) between measured blood pressure and the corresponding 95th-percentile threshold, and the ratio of measured blood pressure to that threshold, each summarized as the maximum across systolic and diastolic components.

### 2.5 Covariates

Covariates were selected on substantive grounds as factors plausibly associated with both central adiposity and blood pressure in this age range, rather than by data-driven selection. The fully adjusted model included age (years, continuous), sex, survey year, BMI (continuous, within the normal range), total energy intake, percentage of energy from fat, percentage of energy from protein, household income per capita (adjusted to a common consumer price index reference year), household size, and a community-level urbanicity index. Dietary variables were derived from three consecutive 24-h recalls collected at the corresponding wave. Height was not entered separately because it is a component of both BMI and WHtR and because the outcome definitions are already height-standardized.

### 2.6 Statistical analysis

Participant characteristics at the first eligible observation were summarized overall and by central-obesity status. Continuous variables are presented as mean (standard deviation) and median (interquartile range), and categorical variables as number (percentage). Group differences at the first eligible observation were compared using Welch t-tests for continuous variables and χ^2^ test for categorical variables.

Primary associations between central obesity and repeated blood-pressure outcomes were estimated using generalized estimating equations (GEE) with a binomial distribution, logit link, exchangeable working correlation structure, and robust sandwich standard errors. Participant identifier was specified as the clustering variable to account for repeated observations from the same child. Four sequential models were fitted: M0: unadjusted model; M1: adjusted for age, sex, and survey year; M2: additionally adjusted for BMI, total energy intake, and percentages of energy from fat and protein; M3: additionally adjusted for household income per capita, household size, and urbanicity index. The fully adjusted M3 model was considered the primary analysis. Associations are reported as odds ratios (ORs) with 95% confidence intervals (CIs). Because central-obesity status and blood-pressure outcomes were measured at the same visit, these models estimate contemporaneous population-average associations.

To aid clinical interpretation, standardized absolute risks under central-obesity and non-central-obesity exposure scenarios were estimated from the fully adjusted GEE models. Absolute risk differences were calculated as the difference between these standardized risks. Approximate population-attributable fractions and event rates per 1,000 person-years were calculated descriptively and are presented as contextual measures rather than causal estimates.

Several supportive analyses were conducted. First, the primary models were repeated after excluding the extreme 1% of SBP and DBP values. Second, analyses were restricted to participants with repeated eligible observations. Third, age-, sex-, and survey-year-adjusted models were estimated. Fourth, WHtR was analyzed as a continuous exposure per 0.05-unit increment. Stratified analyses were performed by sex, age group (7–11 and 12–17 years), urbanicity tertile, and survey period (1993–2000 and 2004–2011). Effect modification was assessed by adding interaction terms between central obesity and sex, age group, or urbanicity to the fully adjusted model. These interaction analyses were considered exploratory. Observed prevalence of above-normal BP and high BP was also summarized across prespecified WHtR categories: <0.40, 0.40-, 0.45-, and ≥0.50.

For exploratory longitudinal analyses, lagged central-obesity status at one survey wave was related to blood-pressure outcomes at the subsequent wave among participants with consecutive observations. Additional descriptive analyses examined exposure patterns across repeated assessments (never, incident, remitting, and persistent central obesity), cumulative central-obesity burden, adjacent-wave state transitions, WHtR change categories, and empirical blood-pressure trajectory patterns. Markov transition models were used to describe observed transition probabilities and projected state distributions. Given the limited number of exposed transitions and persistent central-obesity patterns, these analyses were considered descriptive and exploratory and were not interpreted as causal disease-progression models.

All analyses were performed using R version 4.1.3 (R Foundation for Statistical Computing, Vienna, Austria). Statistical tests were two-sided, and *p* < 0.05 was considered statistically significant for the primary analyses.

## 3 Results

### 3.1 Participant flow and baseline characteristics

After applying the eligibility criteria, 7,469 normal-BMI person-wave observations were included in the descriptive analytic sample, including 323 observations with central obesity (Figure 1A). The distribution of BMI status before restriction and the survey-year prevalence of central obesity within the normal-BMI risk set are presented in Figures 1B,C. The complete-case GEE analysis included 7,192 observations from 4,841 participants, including 317 observations with central obesity (Supplementary Table S1).

**Figure 1:**
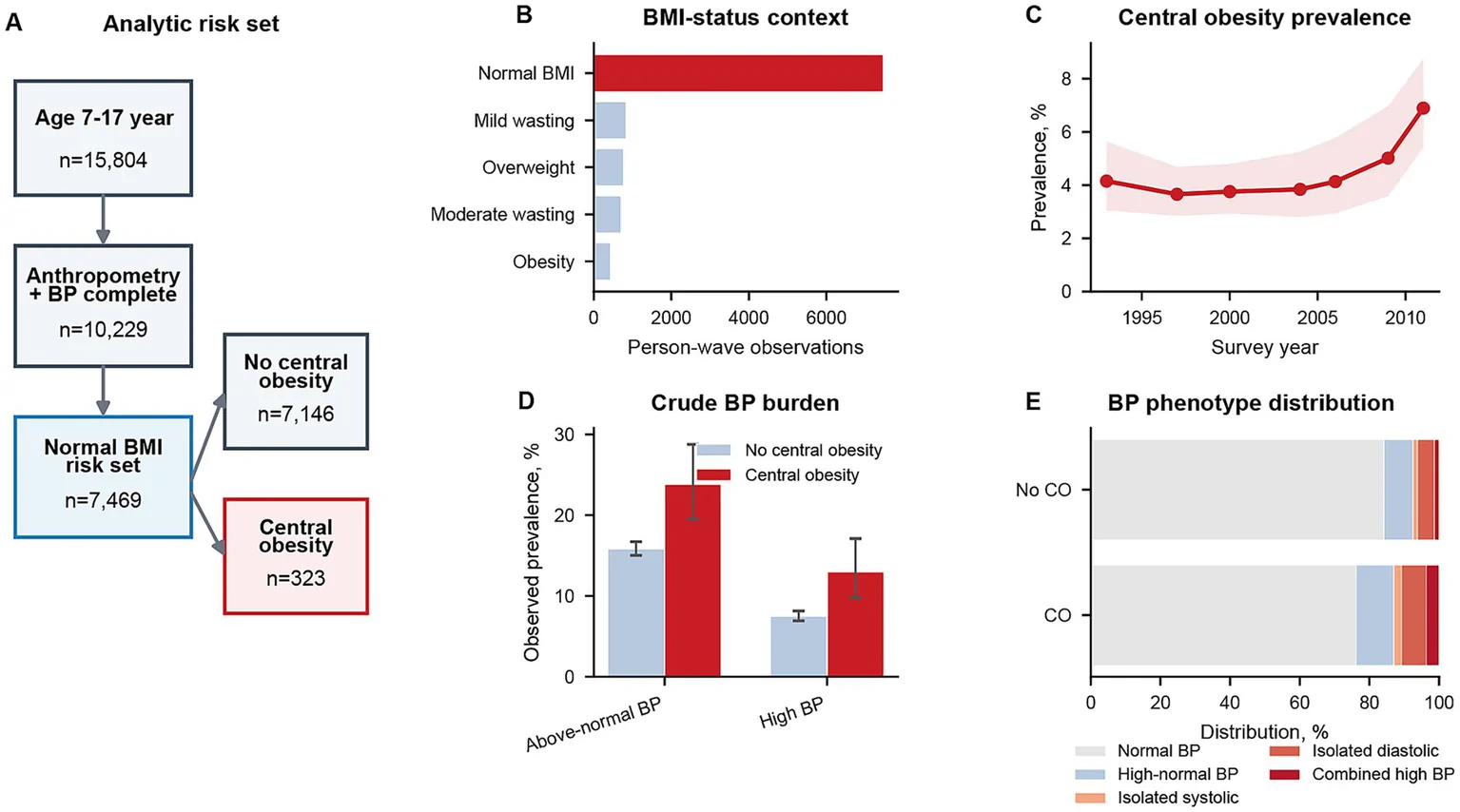
Study population, adiposity context, and observed blood-pressure burden among Chinese children and adolescents with normal BMI. **(A)** Construction of the person-wave analytic risk set. **(B)** Distribution of BMI status before restriction to the normal-BMI analytic risk set. **(C)** Prevalence of central obesity across survey years among normal-BMI observations. **(D)** Crude prevalence of above-normal BP and high BP according to central-obesity status. **(E)** Distribution of BP phenotypes according to central-obesity status.

At first eligible observation, participants with central obesity (*n* = 222) and without (*n* = 4,742) had similar age and weight but, by definition, higher waist circumference (73.42 vs. 60.47 cm) and WHtR (0.53 vs. 0.42), with modestly higher BMI despite both groups falling within the normal range (17.97 vs. 17.17 kg/m^2^) (Table 1). Above-normal blood pressure was present in 20.7% of the central-obesity group versus 14.4% of the non-central-obesity group, and high blood pressure in 13.1% versus 7.1% (Table 1; Figure 1D); the distribution of blood-pressure phenotypes by exposure status is shown in Figure 1E. Dietary intake, household income, household size, and urbanicity were broadly similar between groups (Supplementary Table S2).

**Table 1:** Characteristics of children and adolescents with normal BMI at their first eligible observation, overall and by central-obesity status.

Characteristic	Overall *N* = 4,964	No central obesity *N* = 4,742	Central obesity *N* = 222	*P* value
Age (years), mean (SD)	11.54 (3.04)	11.55 (3.04)	11.46 (3.18)	0.680
Height (cm), mean (SD)	142.62 (16.51)	142.79 (16.38)	138.95 (18.61)	0.003
Weight (kg), mean (SD)	36.18 (11.92)	36.18 (11.84)	36.24 (13.55)	0.948
Waist circumference (cm), mean (SD)	61.05 (8.09)	60.47 (7.44)	73.42 (10.92)	<0.001
BMI (kg/m^2^), mean (SD)	17.21 (2.12)	17.17 (2.10)	17.97 (2.39)	<0.001
Waist-to-height ratio, mean (SD)	0.43 (0.04)	0.42 (0.03)	0.53 (0.03)	<0.001
Systolic BP (mm Hg), mean (SD)	97.45 (12.77)	97.37 (12.70)	98.98 (14.13)	0.097
Diastolic BP (mm Hg), mean (SD)	63.89 (9.46)	63.80 (9.45)	65.75 (9.63)	0.004
Female, No. (%)	2,465 (49.7)	2,367 (49.9)	98 (44.1)	0.093
High BP at survey examination, No. (%)	364 (7.3)	335 (7.1)	29 (13.1)	0.001
Above-normal BP, No. (%)	731 (14.7)	685 (14.4)	46 (20.7)	0.010

Above-normal BP included high-normal BP and high BP. Abbreviations: BMI, body mass index; BP, blood pressure; SD, standard deviation.

### 3.2 Primary associations between central obesity and blood pressure

In the fully adjusted complete-case GEE model, central obesity was associated with higher concurrent odds of above-normal blood pressure (OR 1.43, 95% CI 1.07–1.90, *p* = 0.016) and high blood pressure (OR 1.54, 95% CI 1.07–2.21, *p* = 0.022) (Table 2; Figure 2A). Across the stepwise model progression (Supplementary Table S3), estimates attenuated modestly from the unadjusted model (above-normal BP: OR 1.66; high BP: OR 1.82) to the fully adjusted model, but confidence intervals excluded 1.00 at every step for both outcomes.

**Table 2:** Fully adjusted repeated-measures associations between central obesity and primary blood-pressure outcomes among children and adolescents with normal BMI.

**Outcome**	**Events/observations**	**Central-obesity events/observations**	**Adjusted OR (95% CI)**	**P value**
Above-normal BP	1159/7192	75/317	1.43 (1.07, 1.90)	0.016
High BP at survey examination	554/7192	40/317	1.54 (1.07, 2.21)	0.022

Model adjusted for age, sex, survey year, BMI, total energy intake, fat and protein energy percentages, household income, household size, and urbanicity index. ORs were estimated using population-average generalized estimating equations. Abbreviations: BP, blood pressure; OR, odds ratio; CI, confidence interval.

**Figure 2:**
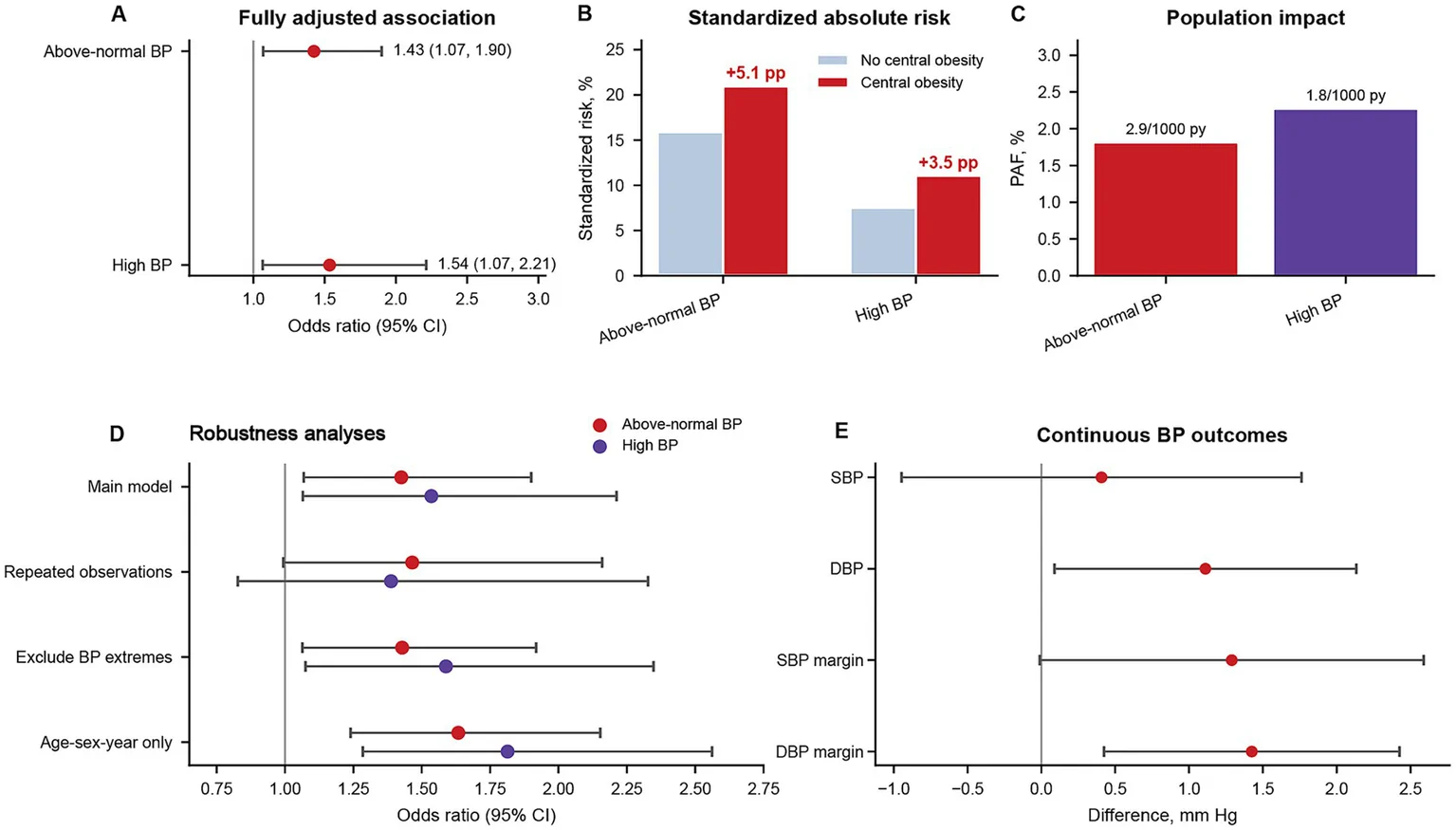
Primary repeated-measures associations, standardized absolute risks, approximate population impact, robustness analyses, and continuous blood-pressure outcomes. **(A)** Fully adjusted population-average odds ratios for the associations of central obesity with above-normal BP and high BP. **(B)** Standardized predicted risks and absolute risk differences comparing central-obesity with non-central-obesity exposure scenarios. **(C)** Approximate population-attributable fractions and event-rate measures for the two primary outcomes. **(D)** Results of supportive robustness analyses, including exclusion of extreme BP values, adjustment for age, sex, and survey year only, and restriction to participants with repeated eligible observations. **(E)** Fully adjusted estimated differences in continuous BP measures associated with central obesity. Abbreviations: BP, blood pressure; CI, confidence interval; DBP, diastolic blood pressure; OR, odds ratio; PAF, population-attributable fraction; py, person-years; SBP, systolic blood pressure.

Standardized absolute risk differences were 5.1 percentage points for above-normal blood pressure and 3.5 percentage points for high blood pressure (Figure 2B; Supplementary Table S5). Approximate population-attributable fractions and event rates (1.8 and 2.9 per 1,000 person-years) are shown in Figure 2C; because central obesity was uncommon in this normal-BMI risk set, population-attributable fractions were small in absolute terms.

### 3.3 Secondary phenotypes and continuous outcomes

Adjusted associations with secondary blood-pressure phenotypes (Supplementary Table S4) were directionally positive but imprecise: high-normal BP (OR 1.18, 95% CI 0.82–1.71), isolated systolic high BP (OR 1.21, 95% CI 0.52–2.80), isolated diastolic high BP (OR 1.42, 95% CI 0.90–2.23), and combined systolic/diastolic high BP (OR 1.91, 95% CI 0.98–3.70); all four confidence intervals included the null value. Continuous SBP/DBP and BP-margin outcomes (Figure 2E) showed a similar pattern of positive but largely imprecise estimates.

### 3.4 Robustness analyses

Estimates for above-normal and high blood pressure were materially unchanged after excluding extreme BP values (ORs 1.43 and 1.59) and were somewhat higher in the age/sex/year-only model (ORs 1.63 and 1.81); estimates in the repeated-observation subgroup were similar in direction but less precise (Supplementary Table S5; Figure 2D). When WHtR was modeled continuously, each 0.05-unit increase yielded ORs of 1.03 (95% CI 0.94–1.14) for above-normal BP and 1.14 (95% CI 0.99–1.30) for high BP, both including the null value (Supplementary Table S10).

### 3.5 Subgroup and interaction analyses

Stratified estimates were directionally consistent with the main analysis across sex, age group, urbanicity tertile, and calendar period, with wider confidence intervals in smaller strata (Supplementary Table S7; Figures 3A–C). Of six exploratory interaction tests (Supplementary Table S6; Figure 3D), only age group showed a nominally significant interaction for high blood pressure (*p* = 0.041), with a stronger association at ages 7–11 years (OR 2.19) than 12–17 years (OR 1.18); the remaining five interaction tests (sex and urbanicity, for both outcomes) had *p* > 0.4.

**Figure 3:**
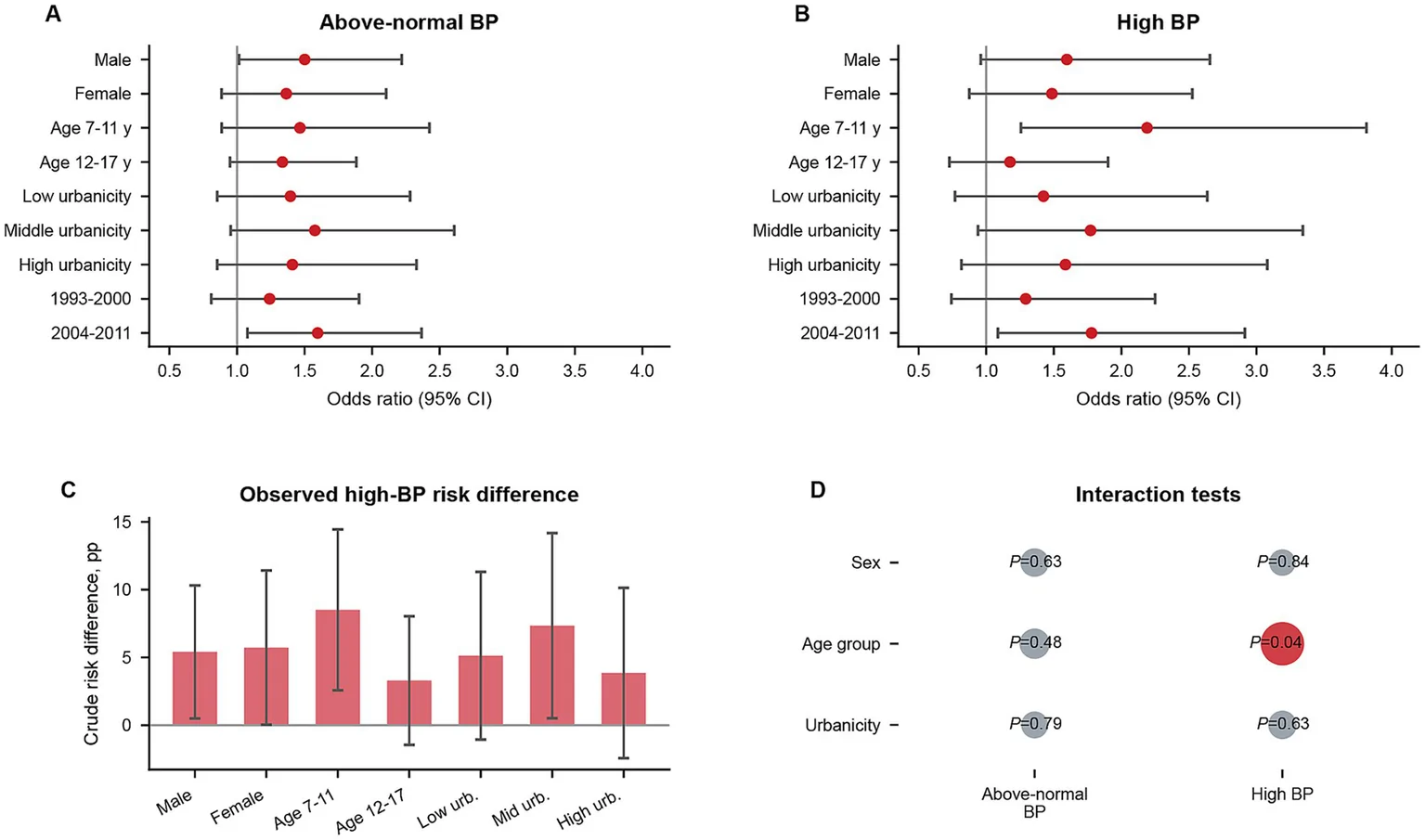
Stratified associations, crude risk differences, and exploratory interaction tests for the association between central obesity and blood-pressure outcomes. **(A)** Fully adjusted odds ratios for above-normal BP according to sex, age group, urbanicity tertile, and survey period. **(B)** Fully adjusted odds ratios for high BP according to the same subgroups. **(C)** Crude risk differences in high BP between participants with and without central obesity within selected clinical strata. **(D)** P values from exploratory interaction tests between central obesity and sex, age group, or urbanicity for above-normal BP and high BP. Abbreviations: BP, blood pressure; CI, confidence interval; OR, odds ratio; pp., percentage points.

### 3.6 WHtR gradient and exploratory longitudinal analyses

Observed prevalence of above-normal and high blood pressure increased across WHtR categories, with the highest prevalence in the ≥0.50 category (Figure 4A). Lagged models (2,446 wave-to-wave transitions, 102 exposed) showed positive but imprecise associations between lagged central obesity and next-wave above-normal BP (OR 1.33, 95% CI 0.80–2.22) and high BP (OR 1.39, 95% CI 0.72–2.68), with continuous next-wave BP differences close to zero (Supplementary Tables S8, S9). Markov-projected burden, transition probabilities, and stationary-state estimates (Figures 4B,C; Supplementary Figure S1; Supplementary Tables S11–S14) and exposure-pattern, cumulative-exposure, and trajectory analyses (Figures 4D–F; Supplementary Figures S2, S3; Supplementary Tables S15–S17) were broadly consistent in direction with the primary findings, but exposed transitions were sparse and confidence intervals for cumulative-exposure contrasts crossed the null value.

**Figure 4:**
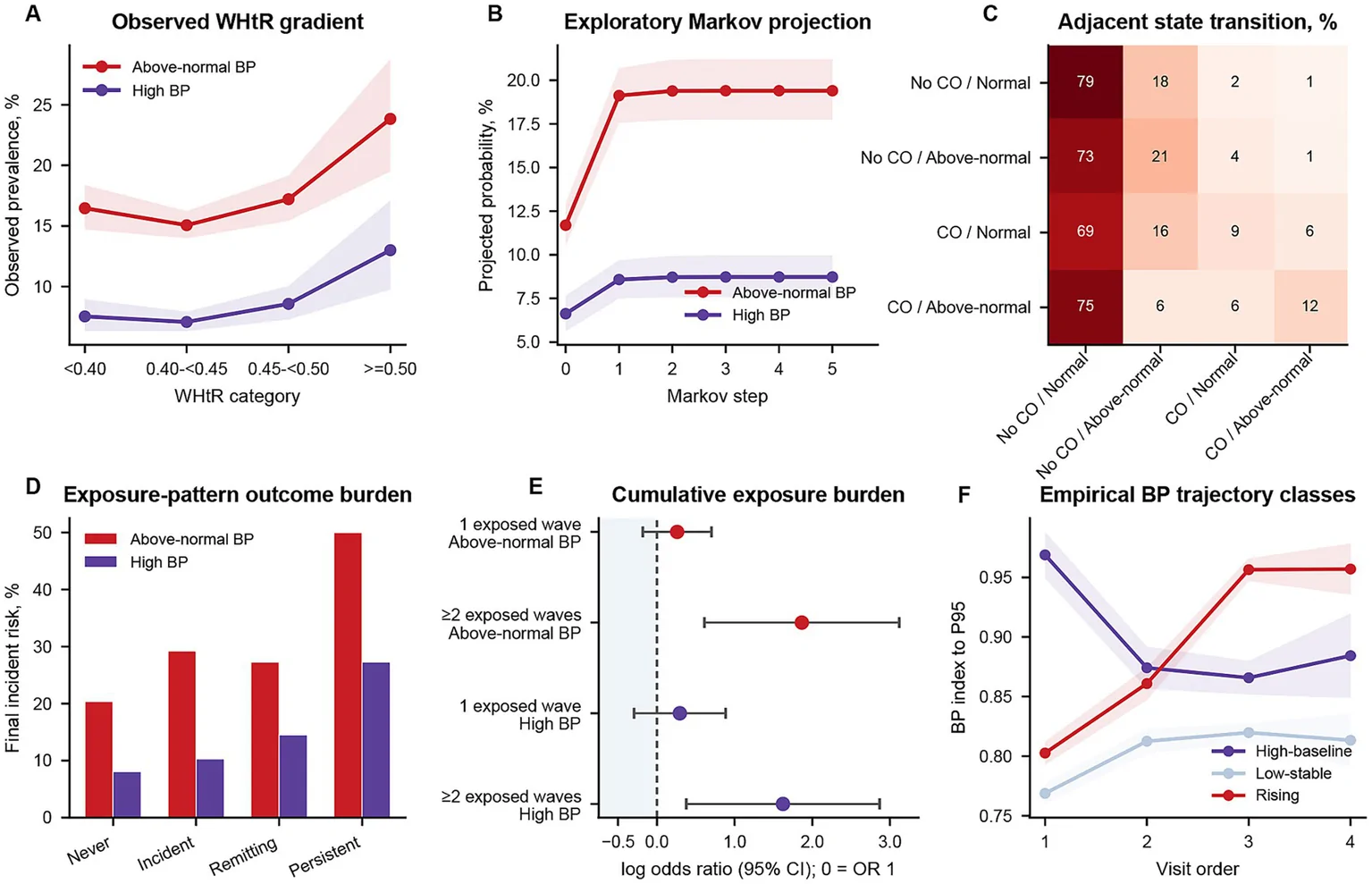
Waist-to-height-ratio gradient and exploratory longitudinal patterns of central obesity and blood-pressure outcomes. **(A)** Observed prevalence of above-normal BP and high BP across prespecified WHtR categories. **(B)** Markov-projected distributions of blood-pressure states over five transition steps. **(C)** Observed adjacent-wave transitions between joint central-obesity and BP states. **(D)** Final-visit prevalence of above-normal BP and high BP according to longitudinal central-obesity exposure patterns. **(E)** Associations of cumulative central-obesity exposure burden with BP outcomes. **(F)** Empirically derived BP trajectory classes across repeated observations. Abbreviations: BP, blood pressure; OR, odds ratio; WHtR, waist-to-height ratio.

## 4 Discussion

In this repeated-measures analysis of Chinese children and adolescents with normal BMI, central obesity defined by WHtR ≥0.50 was associated with higher contemporaneous burden of both above-normal BP and high BP at survey examination. Because exposure and outcome were assessed at the same visit in the primary models, these findings should be interpreted as repeated cross-sectional population-average associations rather than evidence of temporal ordering. In the fully adjusted GEE models, central obesity was associated with 43% higher odds of above-normal BP and 54% higher odds of high BP. On the absolute scale, standardized risk differences were 5.1 percentage points and 3.5 percentage points, respectively. The association remained similar after exclusion of extreme BP values and in alternative model specifications. However, estimates were less precise among participants with repeated observations only, and the continuous WHtR analyses did not reach conventional statistical significance.

Our findings are consistent with evidence indicating that waist-based measures capture cardiometabolic information not fully represented by BMI (24, 25). BMI is useful for assessing body-size status at the population level, but it cannot differentiate abdominal from peripheral adiposity (10). In contrast, waist circumference and WHtR more directly reflect central fat accumulation, which has been associated with cardiometabolic risk factors in children and adolescents (26, 27).

Previous studies have reported that WHtR was associated with cardiovascular risk factors across different populations and age groups (28, 29). Ashwell et al. (30) further indicated that WHtR may be a useful screening measure for cardiometabolic risk, although its predictive performance relative to BMI and waist circumference varied across outcomes and populations. In children, Savva et al. reported that waist circumference and WHtR were more strongly associated than BMI with several cardiovascular risk factors (31). The present study extends this evidence by restricting the analysis to children and adolescents within the normal-BMI range, a group in whom risk related to abdominal adiposity may otherwise remain unrecognized.

The observed association with high BP was stronger among participants aged 7–11 years than among those aged 12–17 years, although this interaction should be interpreted cautiously. The subgroup analyses involved fewer exposed observations, and only one of six interaction tests reached nominal statistical significance. Differences by developmental stage, pubertal maturation, growth velocity, body composition, and the distribution of abdominal adiposity may contribute to age-related variation (32, 33); however, these explanations could not be directly evaluated in the current study.

The categorical WHtR analysis showed the highest observed prevalence of above-normal BP and high BP among participants with WHtR ≥0.50. However, when WHtR was modeled continuously, the association was weaker and confidence intervals included the null value. This difference suggests that the relation may not be adequately represented by a simple linear increase across the full WHtR range. It may also reflect limited precision, a threshold-like association at higher WHtR values, or residual heterogeneity within the normal-BMI population. Accordingly, the present results do not establish WHtR ≥0.50 as a definitive diagnostic threshold for BP risk in Chinese youth. Rather, they indicate that normal-BMI children with WHtR ≥0.50 had a higher observed and adjusted BP burden in this cohort. Future studies with larger numbers of centrally obese normal-BMI participants should investigate potential non-linear associations using flexible approaches, such as restricted cubic splines, and should evaluate whether alternative WHtR thresholds improve risk discrimination.

Several mechanisms may link central adiposity to elevated BP. Visceral and abdominal adipose tissue is metabolically active and may contribute to insulin resistance, low-grade inflammation, endothelial dysfunction, altered adipokine signaling, sympathetic nervous-system activation, and renal sodium retention (34, 35). These processes may influence vascular tone, circulating volume, and BP regulation even when total body mass remains within a conventional normal range (11, 16, 36). The present study did not include direct measures of visceral fat, insulin resistance, inflammatory biomarkers, pubertal status, physical activity, or sodium intake. Therefore, these mechanisms remain biologically plausible explanations rather than directly tested pathways in this cohort.

The findings suggest that BMI alone may not fully characterize adiposity-related BP risk in children and adolescents. WHtR is inexpensive, straightforward to calculate, and may be particularly useful as a complementary anthropometric measure when BMI is normal but waist circumference appears relatively high (27, 37). It should not replace standardized pediatric BMI assessment or repeated BP measurement; rather, it may help identify a subgroup warranting closer attention to BP screening and lifestyle counseling (26, 38). The estimated population-attributable fractions were small, reflecting the relatively low prevalence of central obesity within the normal-BMI analytic sample. Thus, the results should not be interpreted to mean that central obesity accounts for most elevated BP in normal-BMI youth. Its potential value is primarily in identifying individual-level risk that may be missed by BMI-based classification.

Strengths of this study include the repeated-measures design with standardised measurement across waves, the use of national age-, sex-, and height-specific Chinese BP references, adjustment for dietary composition, household economic circumstances, and community urbanicity (variables rarely available in paediatric anthropometric studies), and the reporting of absolute alongside relative estimates.

However, several limitations should also be considered. First, the observational design precludes causal inference. The primary GEE estimates describe population-average associations and do not establish that central obesity causes subsequent BP elevation. Second, BP phenotypes were based on measurements obtained at a single survey examination and should not be interpreted as clinically confirmed hypertension, which requires repeated elevated readings on separate visits. Third, central obesity was uncommon among normal-BMI observations, resulting in limited statistical precision for secondary phenotypes, subgroup analyses, and exploratory longitudinal analyses. Fourth, the lagged, transition, Markov, exposure-pattern, and trajectory analyses were based on irregular survey intervals and sparse exposed transitions; these analyses should therefore be regarded as descriptive and hypothesis-generating. Fifth, the CHNS study waves used in this analysis spanned 1993–2011. Changes in dietary patterns, obesity prevalence, BP measurement practices, and healthcare access may limit direct generalizability to contemporary Chinese youth. Sixth, residual confounding is possible, particularly for physical activity, sedentary behavior, pubertal maturation, dietary sodium, sleep, family history of hypertension, and direct measures of body composition. In addition, we did not formally model the exposure-response shape, because observations with WHtR ≥0.50 represented only 4.4% of the complete-case sample and flexible estimates in this range would have been imprecise and sensitive to modeling choices. Finally, restriction to normal BMI at each examination defined the target risk set but may also have introduced selection-related bias, because BMI is associated with both central adiposity and blood-pressure risk. Accordingly, the findings should be interpreted as associations within a selected normal-BMI person-wave population rather than as effects applicable across the full BMI distribution.

In Conclusion, among Chinese children and adolescents with normal BMI, central obesity defined by WHtR ≥0.50 was associated with higher odds of above-normal BP and high BP at survey examination. These contemporaneous associations indicate that waist-based assessment may provide complementary information beyond BMI for identifying children with an unfavorable BP profile, but do not establish temporal precedence, causality, or incremental screening performance. Confirmation in contemporary prospective cohorts with repeated standardized BP assessment, direct measures of adiposity, and longitudinal cardiometabolic follow-up is warranted.

## Data Availability

The original contributions presented in the study are included in the article/Supplementary material, further inquiries can be directed to the corresponding author/s.
