# Supplementary material for: Central obesity and blood pressure burden among Chinese children and adolescents with normal body mass index: a repeated-measures analysis of the China health and nutrition survey
Source: Front Public Health. 2026 Oct 16;14:1973651. doi: 10.3389/fpubh.2026.1973651 (PMC13719476; doi:10.3389/fpubh.2026.1973651)
Supplement: Supplementary file 1 [file Data_Sheet_1.docx]

**Supplementary Information**

## **Supplementary Table S1. Definition and size of the analytic samples**

| **Dataset** | **Denominator** | **Central-obesity denominator** |
| --- | --- | --- |
| First eligible observation cohort | 4964 participants | 222 central-obesity participants |
| Repeated-observation descriptive cohort | 7469 person-wave observations from 4964 participants | 323 central-obesity person-waves |
| Complete-case GEE sample | 7192 observations from 4841 participants | 317 central-obesity observations |
| Main reason for reduced complete-case sample | 198 observations missing dietary variables; 86 missing household income or household size | categories partly overlapped |

## **Supplementary Table S2. Additional dietary, household, community, and blood-pressure characteristics at the first eligible observation, according to central-obesity status**

| **Characteristic** | **Overall** | **No central obesity** | **Central obesity** | **P value** |
| --- | --- | --- | --- | --- |
| 3-day mean total energy (kcal/d), mean (SD) | 1859.70 (614.87) | 1861.44 (613.30) | 1823.46 (647.16) | 0.393 |
| Carbohydrate energy (%), mean (SD) | 60.58 (12.44) | 60.51 (12.46) | 61.86 (12.11) | 0.106 |
| Fat energy (%), mean (SD) | 26.90 (11.96) | 26.96 (11.97) | 25.53 (11.66) | 0.076 |
| Protein energy (%), mean (SD) | 12.51 (2.93) | 12.50 (2.94) | 12.61 (2.74) | 0.561 |
| Household size, mean (SD) | 4.38 (1.32) | 4.38 (1.32) | 4.44 (1.25) | 0.486 |
| CPI-adjusted household income per capita, mean (SD) | 5950.02 (7507.74) | 5942.73 (7530.17) | 6103.90 (7031.69) | 0.740 |
| Urbanicity index, mean (SD) | 55.95 (19.88) | 56.12 (19.91) | 52.33 (18.93) | 0.004 |
| High-normal BP, No. (%) | 367 (7.4) | 350 (7.4) | 17 (7.7) | 0.878 |

## Note: The table is based on the first eligible observation for each participant (n=4,964). High-normal BP was defined as blood pressure at or above the 90th percentile but below the 95th percentile according to age-, sex-, and height-specific Chinese pediatric reference thresholds. Abbreviations: BP, blood pressure; CPI, consumer price index; SD, standard deviation.

## **Supplementary Table S3. Sequential generalized estimating equation models for the associations of central obesity with above-normal blood pressure and high blood pressure**

| **Outcome** | **Model** | **Observations** | **Events** | **Exposed observations** | **Exposed events** | **OR (95% CI)** | **P value** |
| --- | --- | --- | --- | --- | --- | --- | --- |
| Above-normal BP | M0 | 7469 | 1210 | 323 | 77 | 1.66 (1.27, 2.17) | <0.001 |
| Above-normal BP | M1 | 7469 | 1210 | 323 | 77 | 1.63 (1.24, 2.15) | <0.001 |
| Above-normal BP | M2 | 7271 | 1175 | 319 | 76 | 1.44 (1.08, 1.92) | 0.012 |
| Above-normal BP | M3 | 7192 | 1159 | 317 | 75 | 1.43 (1.07, 1.90) | 0.016 |
| High BP at survey examination | M0 | 7469 | 579 | 323 | 42 | 1.82 (1.29, 2.56) | <0.001 |
| High BP at survey examination | M1 | 7469 | 579 | 323 | 42 | 1.81 (1.28, 2.56) | <0.001 |
| High BP at survey examination | M2 | 7271 | 565 | 319 | 41 | 1.60 (1.12, 2.29) | 0.011 |
| High BP at survey examination | M3 | 7192 | 554 | 317 | 40 | 1.54 (1.07, 2.21) | 0.022 |

Note: Odds ratios were estimated using population-average GEE models. M0 was unadjusted; M1 was adjusted for age, sex, and survey year; M2 was additionally adjusted for BMI, total energy intake, and percentages of energy from fat and protein; and M3 was additionally adjusted for household income per capita, household size, and urbanicity index. Above-normal BP included high-normal BP and high BP. High BP was defined using age-, sex-, and height-specific Chinese pediatric reference thresholds at a single survey examination. Abbreviations: BP, blood pressure; CI, confidence interval; M, model; OR, odds ratio.

## **Supplementary Table S4. Associations of central obesity with secondary blood-pressure phenotypes in the complete-case sample**

| **Outcome** | **Observations** | **Events** | **Exposed observations** | **OR (95% CI)** | **P value** |
| --- | --- | --- | --- | --- | --- |
| High-normal BP | 7192 | 605 | 317 | 1.18 (0.82, 1.71) | 0.381 |
| Isolated systolic high BP | 7192 | 97 | 317 | 1.21 (0.52, 2.80) | 0.654 |
| Isolated diastolic high BP | 7192 | 352 | 317 | 1.42 (0.90, 2.23) | 0.129 |
| Combined systolic/diastolic high BP | 7192 | 105 | 317 | 1.91 (0.98, 3.70) | 0.056 |

Note: The analysis included 7,192 person-wave observations from 4,841 participants. ORs were estimated from fully adjusted population-average GEE models. Models were adjusted for age, sex, survey year, BMI, total energy intake, percentages of energy from fat and protein, household income per capita, household size, and urbanicity index. Isolated systolic high BP and isolated diastolic high BP indicate systolic-only and diastolic-only high BP, respectively; combined systolic/diastolic high BP indicates that both components met the high-BP threshold. Abbreviations: BP, blood pressure; CI, confidence interval; GEE, generalized estimating equations; OR, odds ratio.

## **Supplementary Table S5. Robustness analyses and standardized absolute risk differences for the associations of central obesity with blood-pressure outcomes**

| **Analysis** | **Outcome** | **Observations** | **Events** | **Exposed observations** | **OR (95% CI)** | **P value** | **Risk difference** |
| --- | --- | --- | --- | --- | --- | --- | --- |
| Main M3 complete case | Above-normal BP | 7192 | 1159 | 317 | 1.43 (1.07, 1.90) | 0.016 | 5.1 |
| Repeated-observation subgroup | Above-normal BP | 4271 | 678 | 184 | 1.47 (0.99, 2.16) | 0.054 | 5.4 |
| Excluding SBP/DBP extreme 1% | Above-normal BP | 7051 | 1056 | 310 | 1.43 (1.06, 1.92) | 0.018 | 4.9 |
| Age-sex-year adjusted only | Above-normal BP | 7469 | 1210 | 323 | 1.63 (1.24, 2.15) | <0.001 | 7.4 |
| Main M3 complete case | High BP (>=P95) | 7192 | 554 | 317 | 1.54 (1.07, 2.21) | 0.022 | 3.5 |
| Repeated-observation subgroup | High BP (>=P95) | 4271 | 326 | 184 | 1.39 (0.83, 2.33) | 0.214 | 2.6 |
| Excluding SBP/DBP extreme 1% | High BP (>=P95) | 7051 | 454 | 310 | 1.59 (1.07, 2.35) | 0.020 | 3.3 |
| Age-sex-year adjusted only | High BP (>=P95) | 7469 | 579 | 323 | 1.81 (1.28, 2.56) | <0.001 | 5.3 |

## Note: The main complete-case model was adjusted for age, sex, survey year, BMI, total energy intake, percentages of energy from fat and protein, household income per capita, household size, and urbanicity index. The repeated-observation subgroup was restricted to participants contributing at least two eligible observations. In the extreme-value analysis, the upper and lower 1% of systolic BP and diastolic BP values were excluded. Standardized risk differences are expressed as percentage points and compare predicted risks under central-obesity and non-central-obesity exposure scenarios. Abbreviations: BP, blood pressure; CI, confidence interval; M3, fully adjusted model; OR, odds ratio; SBP, systolic blood pressure.

## **Supplementary Table S6. Exploratory tests of interaction between central obesity and selected participant characteristics for blood-pressure outcomes**

| **Outcome** | **Modifier** | **Central obesity OR in reference stratum** | **Interaction OR (95% CI)** | **P for interaction** |
| --- | --- | --- | --- | --- |
| Above-normal BP | Sex | 1.51 (1.04, 2.21) | 0.87 (0.49, 1.54) | 0.632 |
| Above-normal BP | Age group | 1.64 (1.00, 2.67) | 0.81 (0.45, 1.45) | 0.476 |
| Above-normal BP | Urbanicity | 1.43 (1.07, 1.90) | 1.04 (0.79, 1.36) | 0.786 |
| High BP (>=P95) | Sex | 1.59 (0.96, 2.63) | 0.93 (0.46, 1.90) | 0.841 |
| High BP (>=P95) | Age group | 2.38 (1.39, 4.06) | 0.48 (0.23, 0.97) | 0.041 |
| High BP (>=P95) | Urbanicity | 1.55 (1.08, 2.23) | 1.08 (0.79, 1.49) | 0.628 |

## Note: Interaction terms between central obesity and sex, age group, or urbanicity were entered separately into the fully adjusted GEE models. The central-obesity OR in the reference stratum represents the exposure association in the designated reference category; the interaction OR represents the ratio of central-obesity ORs between comparison and reference strata. Urbanicity was modeled using the prespecified urbanicity measure used in the interaction model. All interaction analyses were exploratory and were interpreted cautiously. Abbreviations: BP, blood pressure; CI, confidence interval; GEE, generalized estimating equations; OR, odds ratio.

## **Supplementary Table S7. Stratified associations of central obesity with above-normal blood pressure and high blood pressure**

| **Outcome** | **Stratifier** | **Stratum** | **Observations** | **Events** | **Exposed observations** | **OR (95% CI)** | **P value** |
| --- | --- | --- | --- | --- | --- | --- | --- |
| Above-normal BP | Sex | Male | 3580 | 619 | 176 | 1.50 (1.02, 2.22) | 0.042 |
| Above-normal BP | Sex | Female | 3612 | 540 | 141 | 1.36 (0.89, 2.10) | 0.159 |
| Above-normal BP | Age group | 7-11 years | 3002 | 312 | 138 | 1.47 (0.89, 2.42) | 0.136 |
| Above-normal BP | Age group | 12-17 years | 4190 | 847 | 179 | 1.34 (0.95, 1.88) | 0.098 |
| Above-normal BP | Urbanicity tertile | Low urbanicity | 2413 | 402 | 118 | 1.40 (0.85, 2.28) | 0.184 |
| Above-normal BP | Urbanicity tertile | Middle urbanicity | 2408 | 356 | 102 | 1.58 (0.95, 2.61) | 0.076 |
| Above-normal BP | Urbanicity tertile | High urbanicity | 2371 | 401 | 97 | 1.41 (0.85, 2.33) | 0.179 |
| Above-normal BP | Period | 1993-2000 | 4027 | 597 | 157 | 1.24 (0.81, 1.90) | 0.321 |
| Above-normal BP | Period | 2004-2011 | 3165 | 562 | 160 | 1.60 (1.08, 2.37) | 0.020 |
| High BP (>=P95) | Sex | Male | 3580 | 256 | 176 | 1.60 (0.96, 2.66) | 0.072 |
| High BP (>=P95) | Sex | Female | 3612 | 298 | 141 | 1.49 (0.88, 2.52) | 0.143 |
| High BP (>=P95) | Age group | 7-11 years | 3002 | 185 | 138 | 2.19 (1.26, 3.81) | 0.006 |
| High BP (>=P95) | Age group | 12-17 years | 4190 | 369 | 179 | 1.18 (0.73, 1.90) | 0.509 |
| High BP (>=P95) | Urbanicity tertile | Low urbanicity | 2413 | 205 | 118 | 1.42 (0.77, 2.64) | 0.261 |
| High BP (>=P95) | Urbanicity tertile | Middle urbanicity | 2408 | 173 | 102 | 1.77 (0.94, 3.34) | 0.077 |
| High BP (>=P95) | Urbanicity tertile | High urbanicity | 2371 | 176 | 97 | 1.59 (0.82, 3.08) | 0.173 |
| High BP (>=P95) | Period | 1993-2000 | 4027 | 290 | 157 | 1.29 (0.74, 2.25) | 0.365 |
| High BP (>=P95) | Period | 2004-2011 | 3165 | 264 | 160 | 1.78 (1.09, 2.91) | 0.022 |

## Note: Models were adjusted for age, sex, survey year, BMI, total energy intake, percentages of energy from fat and protein, household income per capita, household size, and urbanicity index, except for the variable used for stratification where applicable. Stratified analyses were exploratory. Abbreviations: BMI, body mass index; BP, blood pressure; CI, confidence interval; OR, odds ratio.

## **Supplementary Table S8. Exploratory lagged associations between central obesity at one survey wave and blood-pressure outcomes at the subsequent wave**

| **Outcome** | **Transitions** | **Individuals** | **Next-wave events** | **Lagged exposed transitions** | **OR (95% CI)** | **P value** |
| --- | --- | --- | --- | --- | --- | --- |
| Above-normal BP | 2446 | 1886 | 468 | 102 | 1.33 (0.80, 2.22) | 0.275 |
| High BP | 2446 | 1886 | 212 | 102 | 1.39 (0.72, 2.68) | 0.323 |

## Note: Analyses were restricted to consecutive eligible person-wave transitions. Lagged central obesity was defined as WHtR ≥0.50 at the preceding survey wave. ORs compare participants with versus without central obesity at the preceding wave for the specified next-wave blood-pressure outcome. Abbreviations: BP, blood pressure; CI, confidence interval; OR, odds ratio; WHtR, waist-to-height ratio.

## **Supplementary Table S9. Exploratory lagged associations between central obesity at one survey wave and continuous blood-pressure measures at the subsequent wave**

| **Outcome** | **Transitions** | **Lagged exposed transitions** | **Difference (95% CI)** | **P value** |
| --- | --- | --- | --- | --- |
| Next-wave SBP, mmHg | 2446 | 102 | -0.17 (-2.33, 2.00) | 0.88 |
| Next-wave DBP, mmHg | 2446 | 102 | 0.04 (-1.72, 1.80) | 0.964 |
| Next-wave max BP margin to P95, mmHg | 2446 | 102 | 0.40 (-1.29, 2.09) | 0.644 |
| Next-wave max BP/P95 index | 2446 | 102 | 0.01 (-0.01, 0.03) | 0.550 |

## Note: Analyses were restricted to consecutive eligible person-wave transitions. Lagged central obesity was defined as WHtR ≥0.50 at the preceding survey wave. Differences represent the estimated mean difference in the specified next-wave continuous blood-pressure measure comparing lagged central obesity with no lagged central obesity. The maximum BP margin to P95 was defined as the larger of the systolic and diastolic differences between observed BP and the corresponding 95th-percentile threshold. The maximum BP/P95 index was the larger of the systolic and diastolic ratios of observed BP to the corresponding 95th-percentile threshold. Abbreviations: BP, blood pressure; CI, confidence interval; DBP, diastolic blood pressure; P95, age-, sex-, and height-specific 95th-percentile blood-pressure threshold; SBP, systolic blood pressure; WHtR, waist-to-height ratio.

## **Supplementary Table S10. Associations of waist-to-height ratio modeled continuously with above-normal blood pressure and high blood pressure**

| **Outcome** | **Exposure** | **Observations** | **Events** | **OR (95% CI)** | **P value** |
| --- | --- | --- | --- | --- | --- |
| Above-normal BP | WHtR per 0.05 increase | 7192 | 1159 | 1.03 (0.94, 1.14) | 0.525 |
| High BP (>=P95) | WHtR per 0.05 increase | 7192 | 554 | 1.14 (0.99, 1.30) | 0.066 |

## Note: WHtR was modeled continuously per 0.05-unit increase. ORs were estimated from fully adjusted GEE models. Models were adjusted for age, sex, survey year, BMI, total energy intake, percentages of energy from fat and protein, household income per capita, household size, and urbanicity index. Abbreviations: BMI, body mass index; BP, blood pressure; CI, confidence interval; GEE, generalized estimating equations; OR, odds ratio; WHtR, waist-to-height ratio.

**Supplementary Table S11. Observed transition counts and probabilities for joint central-obesity and above-normal blood-pressure states**

| **State at the preceding examination** | **State at the subsequent examination** | **Number of transitions** | **Origin-state denominator** | **Transition probability, % (95% CI)** |
| --- | --- | --- | --- | --- |
| No CO / Normal BP | No CO / Normal BP | 1,683 | 2,126 | 79.2 (77.5, 80.9) |
| No CO / Normal BP | No CO / Above-normal BP | 374 | 2,126 | 17.6 (16.0, 19.2) |
| No CO / Normal BP | CO / Normal BP | 49 | 2,126 | 2.3 (1.7, 2.9) |
| No CO / Normal BP | CO / Above-normal BP | 20 | 2,126 | 0.9 (0.6, 1.4) |
| No CO / Above-normal BP | No CO / Normal BP | 202 | 277 | 72.9 (67.9, 78.0) |
| No CO / Above-normal BP | No CO / Above-normal BP | 59 | 277 | 21.3 (16.6, 26.7) |
| No CO / Above-normal BP | CO / Normal BP | 12 | 277 | 4.3 (2.2, 6.9) |
| No CO / Above-normal BP | CO / Above-normal BP | 4 | 277 | 1.4 (0.4, 2.9) |
| CO / Normal BP | No CO / Normal BP | 59 | 86 | 68.6 (59.3, 77.9) |
| CO / Normal BP | No CO / Above-normal BP | 14 | 86 | 16.3 (9.3, 24.4) |
| CO / Normal BP | CO / Normal BP | 8 | 86 | 9.3 (3.5, 16.3) |
| CO / Normal BP | CO / Above-normal BP | 5 | 86 | 5.8 (1.2, 11.6) |
| CO / Above-normal BP | No CO / Normal BP | 12 | 16 | 75.0 (50.0, 93.8) |
| CO / Above-normal BP | No CO / Above-normal BP | 1 | 16 | 6.25 (0.0, 18.8) |
| CO / Above-normal BP | CO / Normal BP | 1 | 16 | 6.25 (0.0, 18.8) |
| CO / Above-normal BP | CO / Above-normal BP | 2 | 16 | 12.5 (0.0, 31.2) |

Note: Central obesity was defined as WHtR ≥0.50. Above-normal BP was defined as systolic or diastolic BP at or above the age-, sex-, and height-specific 90th percentile, including high BP. “Normal BP” in this table denotes absence of above-normal BP. The estimates describe changes between observed examinations and should not be interpreted as causal effects or clinical diagnoses of hypertension. Abbreviations: BP, blood pressure; CI, confidence interval; CO, central obesity; WHtR, waist-to-height ratio.

**Supplementary Table S12. Observed transition counts and probabilities for joint central-obesity and high-blood-pressure states**

| **State at the preceding examination** | **State at the subsequent examination** | **Number of transitions** | **Origin-state denominator** | **Transition probability, % (95% CI)** |
| --- | --- | --- | --- | --- |
| No CO / Normal BP | No CO / Normal BP | 1,999 | 2,248 | 88.9 (87.6, 90.2) |
| No CO / Normal BP | No CO / High BP | 173 | 2,248 | 7.7 (6.6, 8.8) |
| No CO / Normal BP | CO / Normal BP | 69 | 2,248 | 3.1 (2.4, 3.8) |
| No CO / Normal BP | CO / High BP | 7 | 2,248 | 0.3 (0.1, 0.5) |
| No CO / High BP | No CO / Normal BP | 124 | 155 | 80.0 (73.5, 85.8) |
| No CO / High BP | No CO / High BP | 22 | 155 | 14.2 (9.0, 20.0) |
| No CO / High BP | CO / Normal BP | 7 | 155 | 4.5 (1.3, 8.4) |
| No CO / High BP | CO / High BP | 2 | 155 | 1.3 (0.0, 3.2) |
| CO / Normal BP | No CO / Normal BP | 70 | 91 | 76.9 (68.1, 85.7) |
| CO / Normal BP | No CO / High BP | 7 | 91 | 7.7 (2.2, 13.2) |
| CO / Normal BP | CO / Normal BP | 11 | 91 | 12.1 (5.5, 18.7) |
| CO / Normal BP | CO / High BP | 3 | 91 | 3.3 (0.0, 7.7) |
| CO / High BP | No CO / Normal BP | 9 | 11 | 81.8 (54.5, 100.0) |
| CO / High BP | No CO / High BP | 0 | 11 | 0.0 (0.0, 0.0) |
| CO / High BP | CO / Normal BP | 1 | 11 | 9.1 (0.0, 27.3) |
| CO / High BP | CO / High BP | 1 | 11 | 9.1 (0.0, 27.3) |

Note: The zero-width interval for the zero-count cell is reproduced from the analysis output and should not be interpreted as establishing that the population transition probability is zero. These estimates are exploratory and are especially imprecise for origin states with few transitions. Abbreviations: BP, blood pressure; CI, confidence interval; CO, central obesity; WHtR, waist-to-height ratio.

**Supplementary Table S13. Markov-projected prevalence of above-normal blood pressure and high blood pressure across transition steps 0–5**

| **BP outcome** | **Transition step** | **Projected prevalence, % (95% CI)** |
| --- | --- | --- |
| Above-normal BP | 0 | 11.7 (10.5, 12.9) |
| Above-normal BP | 1 | 19.1 (17.6, 20.7) |
| Above-normal BP | 2 | 19.4 (17.7, 21.2) |
| Above-normal BP | 3 | 19.4 (17.7, 21.2) |
| Above-normal BP | 4 | 19.4 (17.7, 21.2) |
| Above-normal BP | 5 | 19.4 (17.7, 21.2) |
| High BP | 0 | 6.6 (5.6, 7.7) |
| High BP | 1 | 8.6 (7.5, 9.7) |
| High BP | 2 | 8.7 (7.6, 9.9) |
| High BP | 3 | 8.7 (7.6, 10.0) |
| High BP | 4 | 8.7 (7.6, 10.0) |
| High BP | 5 | 8.7 (7.6, 10.0) |

Note: Projections were obtained by iteratively applying the corresponding observed four-state transition matrix to the initial joint-state distribution. Step 0 represents the observed origin-state distribution in the descriptive transition sample. At each step, the projected prevalence of the BP outcome is the sum of the probabilities of the two joint states with that outcome, irrespective of central-obesity status. A transition step is a model iteration and does not represent a fixed number of calendar years, because examination intervals varied. Abbreviations: BP, blood pressure; CI, confidence interval.

**Supplementary Table S14. Initial and stationary distributions of joint central-obesity and blood-pressure states**

| **BP transition chain** | **Joint state** | **Initial probability, %** | **Stationary probability, % (95% CI)** |
| --- | --- | --- | --- |
| Above-normal BP | No CO / Normal BP | 84.87 | 77.7 (75.7, 79.5) |
| Above-normal BP | No CO / Above-normal BP | 11.06 | 18.1 (16.5, 19.7) |
| Above-normal BP | CO / Normal BP | 3.43 | 2.9 (2.2, 3.7) |
| Above-normal BP | CO / Above-normal BP | 0.64 | 1.3 (0.8, 2.0) |
| High BP | No CO / Normal BP | 89.74 | 87.7 (86.3, 89.1) |
| High BP | No CO / High BP | 6.19 | 8.2 (7.1, 9.4) |
| High BP | CO / Normal BP | 3.63 | 3.5 (2.8, 4.4) |
| High BP | CO / High BP | 0.44 | 0.5 (0.3, 0.9) |

Note: Each row reports the probability of a joint central-obesity–BP state, not the conditional probability of the BP outcome within a central-obesity group. Thus, the stationary prevalence of above-normal BP combines the “No CO / Above-normal BP” and “CO / Above-normal BP” states; the stationary prevalence of high BP similarly combines the two high-BP states. “Normal BP” denotes absence of the outcome used to define the respective chain. Abbreviations: BP, blood pressure; CI, confidence interval; CO, central obesity.

**Supplementary Table S15. Distribution of central-obesity exposure patterns and follow-up characteristics among participants with repeated eligible observations**

| **Central-obesity exposure pattern** | **Participants, n** | **Participants, %** | **Mean number of eligible observations** | **Mean follow-up duration, years** |
| --- | --- | --- | --- | --- |
| Never | 1,758 | 91.47 | 2.30 | 4.31 |
| Incident | 75 | 3.90 | 2.31 | 4.28 |
| Remitting | 76 | 3.95 | 2.28 | 4.32 |
| Persistent | 13 | 0.68 | 2.23 | 3.69 |

Note: Exposure-pattern categories refer to central-obesity status at the first and final eligible examinations: never, absent at both examinations; incident, absent at the first examination and present at the final examination; remitting, present at the first examination and absent at the final examination; and persistent, present at both examinations. Central obesity was defined as WHtR ≥0.50. Follow-up duration was measured between the first and final eligible examinations. Counts represent participants rather than person-wave observations. Abbreviations: WHtR, waist-to-height ratio.

**Supplementary Table S16. Adjusted associations of central-obesity exposure patterns with blood-pressure outcomes at the final eligible examination**

| **Final-examination BP outcome** | **Central-obesity exposure pattern** | **Adjusted OR (95% CI)** | **P value** |
| --- | --- | --- | --- |
| Above-normal BP | Never | 1.00 (reference) | — |
| Above-normal BP | Incident | 1.32 (0.72, 2.43) | 0.37 |
| Above-normal BP | Remitting | 1.54 (0.83, 2.85) | 0.17 |
| Above-normal BP | Persistent | 6.28 (1.54, 25.69) | 0.011 |
| High BP | Never | 1.00 (reference) | — |
| High BP | Incident | 1.01 (0.39, 2.58) | 0.988 |
| High BP | Remitting | 2.07 (1.02, 4.19) | 0.043 |
| High BP | Persistent | 5.05 (1.26, 20.22) | 0.022 |

Note: Models adjusted for age, sex, BMI, total energy intake, percentages of energy from fat and protein, inverse-hyperbolic-sine-transformed income, household size, and urbanicity index at the first eligible examination, together with follow-up duration and baseline survey wave. Abbreviations: BMI, body mass index; BP, blood pressure; CI, confidence interval; OR, odds ratio.

**Supplementary Table S17. Adjusted associations of cumulative central-obesity exposure burden with blood-pressure outcomes at the final eligible examination**

| **Final-examination BP outcome** | **Number of central-obesity-exposed waves** | **Adjusted OR (95% CI)** | **P value** |
| --- | --- | --- | --- |
| Above-normal BP | 0 | 1.00 (reference) | — |
| Above-normal BP | 1 | 1.30 (0.83, 2.02) | 0.249 |
| Above-normal BP | ≥2 | 6.46 (1.84, 22.64) | 0.004 |
| High BP | 0 | 1.00 (reference) | — |
| High BP | 1 | 1.34 (0.75, 2.42) | 0.326 |
| High BP | ≥2 | 5.07 (1.46, 17.61) | 0.011 |

Note: Cumulative central-obesity exposure burden was categorized as 0, 1, or ≥2 eligible examinations with WHtR ≥0.50, with 0 exposed examinations as the reference category. Models adjusted for baseline age, sex, BMI, total energy intake, percentages of energy from fat and protein, inverse-hyperbolic-sine-transformed income, household size, and urbanicity index, together with follow-up duration and baseline survey wave. Abbreviations: BMI, body mass index; BP, blood pressure; CI, confidence interval; OR, odds ratio; WHtR, waist-to-height ratio.

**Supplementary Figure S1. Exploratory Markov transition matrices, projected blood-pressure burden, and stationary-state distributions among children and adolescents with repeated eligible observations**

Note: (A) Observed adjacent-wave transition probabilities for the joint central-obesity and above-normal blood-pressure (BP) states. (B) Observed adjacent-wave transition probabilities for the joint central-obesity and high-BP states. Joint states were defined by central-obesity status and the corresponding BP outcome at each eligible survey wave. (C) Markov-projected prevalence of above-normal BP and high BP over five transition steps, based on the observed transition matrices. (D) Estimated stationary distributions of BP states for the above-normal-BP and high-BP transition chains. Central obesity was defined as a waist-to-height ratio (WHtR) ≥0.50. Above-normal BP included high-normal BP and high BP; high BP was defined using age-, sex-, and height-specific Chinese pediatric reference thresholds at the survey examination. Abbreviations: BP, blood pressure; CO, central obesity; WHtR, waist-to-height ratio.

**Supplementary Figure S2. Exploratory analyses of central-obesity exposure patterns, cumulative exposure burden, lagged exposure, and blood-pressure trajectory classes**

Note: (A) Distribution of central-obesity exposure patterns among participants with repeated eligible observations, categorized as never, incident, remitting, or persistent central obesity. (B) Observed final-visit prevalence of above-normal BP and high BP according to central-obesity exposure pattern. (C) Adjusted associations of central-obesity exposure patterns with final blood-pressure outcomes. (D) Associations of cumulative central-obesity burden, defined by the number of exposed waves, with above-normal BP and high BP. (E) Lagged associations of central obesity at the preceding wave with next-wave blood-pressure outcomes. (F) Empirically derived blood-pressure trajectory classes based on the BP/P95 index across visit order.

**Supplementary Figure S3. Exploratory joint-state transitions, central-obesity change patterns, and waist-to-height-ratio changes in relation to next-wave blood-pressure outcomes**

Note: (A) Baseline-to-final joint-state counts defined by central-obesity status and above-normal BP status. (B) Adjacent-wave joint-state transition counts among central-obesity and blood-pressure states. (C) Associations of changes in central-obesity status, persistent, incident, and remitting central obesity, with next-wave above-normal BP and high BP. (D) Observed next-wave blood-pressure risk according to categories of change in WHtR between adjacent survey waves. Abbreviations: BP, blood pressure; CI, confidence interval; CO, central obesity; OR, odds ratio; WHtR, waist-to-height ratio.
